# A real-time system to report abnormal events involving staff in a nuclear medicine therapy unit

**DOI:** 10.1093/rpd/ncad098

**Published:** 2023-05-24

**Authors:** Giorgia Stendardo, Cristina Nuccetelli, Sveva Grande, Alessandra Palma, Gennaro Venoso, Carmine Zicari, Claudio Andenna, Giuseppe Frau, Ilaria Bonanno, Valeria Landoni, Rosa Sciuto, Vicente Bruzzaniti, Bartolomeo Cassano, Giuseppe Iaccarino, Federica Murtas, Cristina Canzi, Felicia Zito, Paola Fattibene

**Affiliations:** Istituto Superiore di Sanità, Core Facilities, Rome 00161, Italy; Istituto Superiore di Sanità, National Center for Radiation Protection and Computational Physics, Rome 00161, Italy; Istituto Superiore di Sanità, National Centre for Innovative Technologies in Public Health, Rome 00161, Italy; Istituto Superiore di Sanità, National Centre for Innovative Technologies in Public Health, Rome 00161, Italy; Istituto Superiore di Sanità, National Center for Radiation Protection and Computational Physics, Rome 00161, Italy; National Institute for Insurance against Accidents at Work, Department of Technological Innovations and Safety of Plants, Products and Anthropics settlements, Rome 00153, Italy; National Institute for Insurance against Accidents at Work, Department of Technological Innovations and Safety of Plants, Products and Anthropics settlements, Rome 00153, Italy; Deep Blue Srl, Rome 00185, Italy; Deep Blue Srl, Rome 00185, Italy; IRCCS Regina Elena National Cancer Institute, Department of research and advanced technology, Rome 00144, Italy; IRCCS Regina Elena National Cancer Institute, Department of research and advanced technology, Rome 00144, Italy; IRCCS Regina Elena National Cancer Institute, Department of research and advanced technology, Rome 00144, Italy; IRCCS Regina Elena National Cancer Institute, Department of research and advanced technology, Rome 00144, Italy; IRCCS Regina Elena National Cancer Institute, Department of research and advanced technology, Rome 00144, Italy; IRCCS Regina Elena National Cancer Institute, Department of research and advanced technology, Rome 00144, Italy; Fondazione IRCCS Ca’ Granda Ospedale Maggiore Policlinico, Milan 20122, Italy; Fondazione IRCCS Ca’ Granda Ospedale Maggiore Policlinico, Milan 20122, Italy; Istituto Superiore di Sanità, Core Facilities, Rome 00161, Italy

## Abstract

A system for internal and voluntary reporting of abnormal events in a Nuclear Medicine Therapy Unit is described. This system is based on the Internet of Things and is composed of an application for mobile devices and a wireless network of detectors. The application is addressed to healthcare professionals and is intended to be a user-friendly tool to make the reporting procedure little laborious. The network of detectors allows for a real-time measurement of the dose distribution in the patient’s room. The staff was involved in all stages, from the design of the dosimetry system and mobile application up to their final testing. Face-to-face interviews were carried out with 24 operators in different roles in the Unit (radiation protection experts, physicians, physicists, nuclear medicine technicians and nurses). The preliminary results of the interviews and the current state of development of the application and the detection network will be described.

## Introduction

In recent years, an increasing attention has been paid to the prevention and learning from accidental and unintentional exposure in all medical radiation exposure practices, starting with radiotherapy and then moving on to brachytherapy, interventional radiology, and Nuclear Medicine Therapy (NMT)^(1–3)^.

The IAEA and the European Council, in their respective Basic Safety Standards^(4, 5)^ (BSS), have included requirements for both preventing and managing unintended and accidental medical exposures, as well as investigating the circumstances when such exposures occur, in order to learn and improve prevention. The EU-BSS sets up requirements for record keeping and analysis and for reporting to competent authorities in case of significant events^(5, 6)^. The IAEA and BSS requirements apply to events with significant mis-exposures to patients. However, incident reporting can provide a basis for analysis, preparation of strategies and actions aimed at correction, improvement, and prevention of re-occurrences in the future also in minor events, as recommended by the Bonn Call for Actions^(3)^. In its Action 7 (‘Improve prevention of medical radiation incidents and accidents’), the Bonn Call for Action recommends implementing and supporting voluntary and educational safety reporting systems with the purpose of learning from the experience of safety-related events in medical uses of radiation, with an emphasis on brachytherapy, interventional radiology and therapeutic nuclear medicine, in addition to external beam radiotherapy. As this kind of reporting is voluntary and for internal use within the organisation, there are no standards for it; existing systems vary in purpose, type and use of information collected, level of confidentiality and feedback to reporters^(7)^.

Although the above-mentioned recommendations focus on the exposure of patients, in some radiological practices (i.e. interventional radiology, brachytherapy and nuclear medicine) incidents may lead to potential harm both to patients and workers. In particular, nuclear medicine practices involve the use of unsealed sources, and accidents may lead to unintended exposure to patients, workers, the public or the environment. Incidents in nuclear medicine imaging and therapy represent about 20% of the total of reported medical radiation events, according to different literature sources^(8, 9)^.

The present work will show preliminary results of an internal reporting system for events that may occur in a NMT Unit with shielded rooms. As the work is funded by the Italian National Institute for Insurance against Accidents at Work (INAIL, Research Sector), it will be aimed at setting up an incident reporting system to be used in case of events involving patients or workers, such as minor accidents or near misses, but also in the case of non-conformities, technical failures or when a dosemeter is lost, forgotten or malfunctioning.

An Internet of Things (IoT) system, based on an application for mobile devices and a wireless network of detectors, is under design and will be hereafter referred to as the SIREN project. In this context, IoT refers to a distributed network of physical objects that are capable of sensing radiation, and able to communicate with each other and computers ^(10)^. The reason behind the choice of an app lies in the intention to create a tool for easy and quick reporting, ideally in real time, in order to minimise inaccuracies that may occur when the report is made some time after the event in paper form or by email, like the errors due, for example, to doubts about the dynamics of the event or to forgetting details. The involvement of the staff was aimed at listening to their experiences and specific needs and collecting their feedback on possible solutions. This seemed to be a necessary step to increase the perceived usefulness of reporting or reduce the fear of the consequences^(9, 11)^. Finally, the purpose of the wireless network of detectors is to record the dose at different locations in the shielded patient room to highlight abnormal values.

A NMT Unit with shielded rooms was chosen because the number of variables is smaller than, for instance, those of an imaging department. In fact, patients are hospitalised in the shielded rooms for several days, foreseeable paths are followed, and a smaller and defined team of operators is involved. This was a clear advantage for a pilot project, despite the undoubtful argument that the annual dose to the staff in this department is expected to be at most 5 mSv^(12, 13)^. Once tested, the system might be extended to other radiological practices, such as interventional radiology or nuclear medicine imaging, that may involve higher dose levels or more frequent accidental or unintended events.

## Methods

### Involvement of the operators and interviews

The operators to be involved in the working group were identified on the basis of the time dedicated to care activities, the diversification of activities and the availability compatible with the regular performance of clinical activities. Exposed category A and B workers and medical and paramedical staff were included. Participation was voluntary. Face-to-face meetings were held to collect opinions of the operators about critical issues in the current system and suggestions for improvement. Procedures for privacy compliance have been followed.

### Application for mobile devices and expert dashboard

The SIREN app is structured in two main parts: a mobile application for data collection and a web-based administration dashboard for consulting the collected data. The app was developed by adopting an Agile methodology, organised in small iterative cycles (sprints), which allows to introduce new requirements even at advanced stages of development, and aims to release working prototypes at short intervals. The mobile app was developed using the Flutter framework of Google^(14)^, which allows the creation of both Android and iOS versions.

### Nuclear medicine facility and shielded room

The system is planned to be tested in two nuclear medicine facilities: the radiometabolic therapy unit of the Nuclear Medicine Department at IRCCS Regina Elena National Cancer Institute (IFO), and the Nuclear Medicine UOC at IRCCS Ca’ Granda Ospedale Maggiore Policlinico (PMI). At IFO, there are eight shielded rooms for inpatients under therapies with ^131^I, ^177^Lu and ^90^Y that are carried out in a protected environment. The shielded room used for testing has one bed and the drains connected to the system for the collection and decay of radioactive liquid biological waste, as well as air conditioning, sampling and expulsion systems.

The PMI is not currently equipped with shielded rooms. Therapies are mostly performed using ^131^I for the outpatient treatment of benign thyroid diseases and by the local administration of ^90^Y glass microspheres to treat hepatic tumors, which do not require the hospitalisation in a shielded room.

### Dose computational tools

To evaluate the type, number and placement of the detectors, the dose to the operator was estimated with RESRAD-BUILD code for ^131^I, in scenarios of routine conditions and some potential abnormal events.

The RESRAD family of codes was developed at Argonne National Laboratory^(15)^. RESRAD-BUILD is a computer deterministic code (based on the point-kernel method) designed to assess the radiation exposure of a human receptor in a contaminated building or a building housing contaminated furniture or equipment. The programme enables to simulate a room and place up to 10 operators in it, and the output is expressed in terms of the effective dose. It has a user-friendly interface, with up to 10 sources allowed and takes up a short computational time.

For the purpose of comparing the results with other experimental or computational data, the effective dose was converted into ambient dose equivalent^(16)^. The patient as a source of gamma radiation was simulated by small volume sources of ^131^I (1 cm^3^ in the stomach immediately after the swallowing and 16 cm^3^ when accumulated in the thyroid), shielded by a water layer. Due to the gamma line energy of ^131^I (364 keV), changes of the layer thickness from 0 to 5 cm did not have a significant impact on the effective doses calculated.

### Dose rate measurements

At PMI dose rate measurements from patients treated with ^90^Y were performed 1 hour after the administration with a calibrated hand-held pancake GM tube at 0.5 and 1 m from the right side of the patients, who were lying down. The same device was used for the front measurements of patients treated with ^131^I for benign thyroid diseases 2.5 hours after administration, when patients were leaving the diagnostic department after a short observation period.

At IFO, dose rate measurements from patients treated with ^90^Y and ^131^I were performed with a Fluke Biomedical ion chamber 1 hour after the administration at 1 m. During the measurement, the ^90^Y-treated patient lays on the bed, whereas the ^131^I-treated patient stands in front of the detector. The radioactivity measurement of the ^177^Lu-treated patients is performed with an AtomLab 950 Thyroid Uptake System.

## Results

### Outcomes of the interviews

The interviews were carried out by two experts in Human Factors to representative operators bearing different roles in the NMT Unit: three nurses/head nurses; two technicians/technical managers; two healthcare assistants; three medical physicists; one physician and one qualified expert in radiation protection.

The face-to-face interviews were conducted in a semi-structured manner and lasted approximately 60 minutes. The topics covered included the following:

- personal data and routine activities: description of one’s role, experience in the department and general educational background;
- protective and dose monitoring devices that are used: opinions on advantages and disadvantages of passive dosemeters;
- situations perceived as having a particular risk of exposure;
- procedures/actions to be taken in case of accidents and abnormal events, including near misses.

The analysis of the interviews, although not immediately applicable to contexts different from the one considered, revealed practical aspects that are useful for the design of a reporting tool. On the whole, a good safety culture was shown, with a broad awareness of the general risk arising from errors in the handling of radioactive substances, regardless of the role of the operator. Instead, a different perception, according to the role/experience, was found when it comes to the risk related to specific substances, circumstances and processing steps, but also to the possibility of minor events/anomalies that may affect the exposure, such as prolonged failures in automatic fractionators or minor spills of radioactive material. The aspects considered challenging were the following: a lack of records; an informal, poorly-structured transfer of information on minor events/anomalies; a need for more accurate/continuous information on the received doses, even in cases where they do not exceed or do not approach the threshold values. No critical issues related to the use of current dosemeters (i.e. devices being forgotten or exchanged, etc.) were detected, and it was stated that their use did not hinder the daily work of any of the roles.

### App interface and reporting workflow

**Figure 1:**
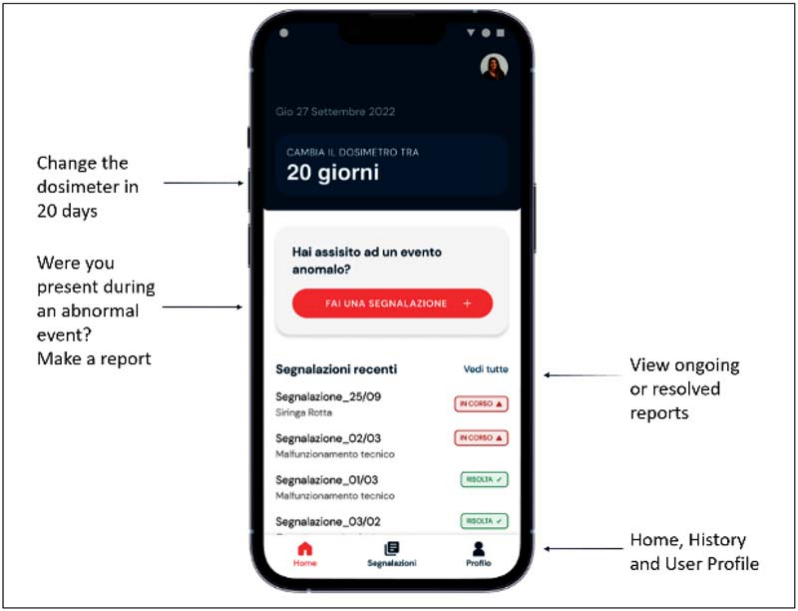
SIREN app interface. The app is in Italian language and the translation of the text in the homepage is shown.

The SIREN app interface is shown in Figure 1. In the first screen, it is possible to add an incident report, view the most recent reports added by other operators (live monitoring) and read warnings and suggestions about the radiation protection surveillance (i.e. the date of the first upcoming medical visit or when the personal dosemeter has to be changed).

User accounts are created by the system manager, and the application is available only to the hospital staff.

Access to the app is granted by means of a user log-in. There will be two types of users: regular users, who can report, read messages, access the live monitoring and receive reminders about radiation protection and health surveillance deadlines; super-users, who have the privilege to edit and share the reports with the staff.

The structure of the app is based on the experiences learned from literature^(7)^. When an event occurs, a new report can be opened by the person present at the event (the reporter). Other staff involved have the possibility to add data and observations. The reporter is asked to add data in two steps:

(1) incident type from a scroll menu, location and time;
(2) narrative description of the event in the form of free text, including the perceived causes and the immediate remedial measures that have been put in place. Photographs and documents can be uploaded.

Once the report has been saved, it is managed by a person with a role of responsibility (with app superuser’s privileges), who can vary depending on the type of event and on where this happened, i.e. the radiation protection expert, the head nurse, the chief technician. Immediately after the report has been received, the person in charge can

- review the event description given by the reporter, which can be integrated, where necessary, after further enquiring from the reporter and the staff involved;
- decide whether the event is relevant and is worth being made public to the whole staff via the app itself.

At a later stage, other information will be able to be uploaded:

- description of the remedial measures;
- data analysis;
- upload of the learned lessons, organisational changes or new procedures, if any.

**Figure 2:**
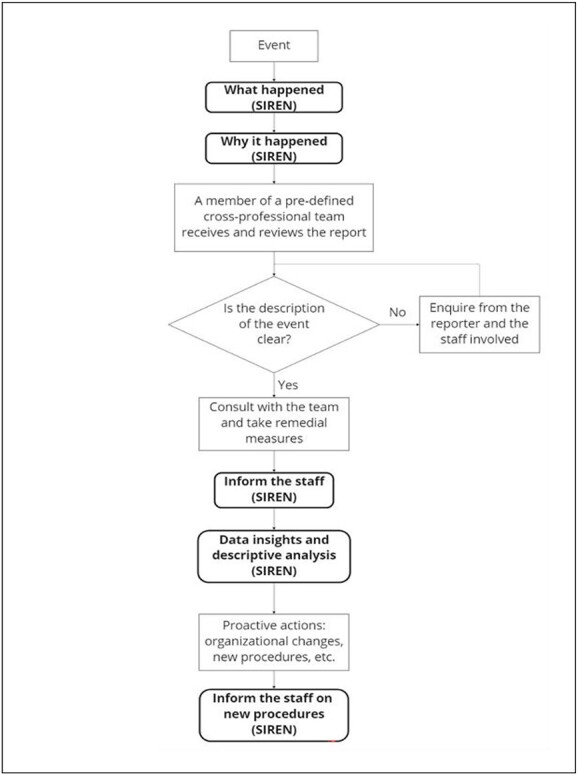
Internal reporting workflow. The boxes in black and bold refer to the steps in which the SIREN application can be used.

The diagram in Figure 2 shows the internal reporting process, highlighting the phases when the SIREN app comes into play.

### Dose estimate in selected scenarios

**Figure 3:**
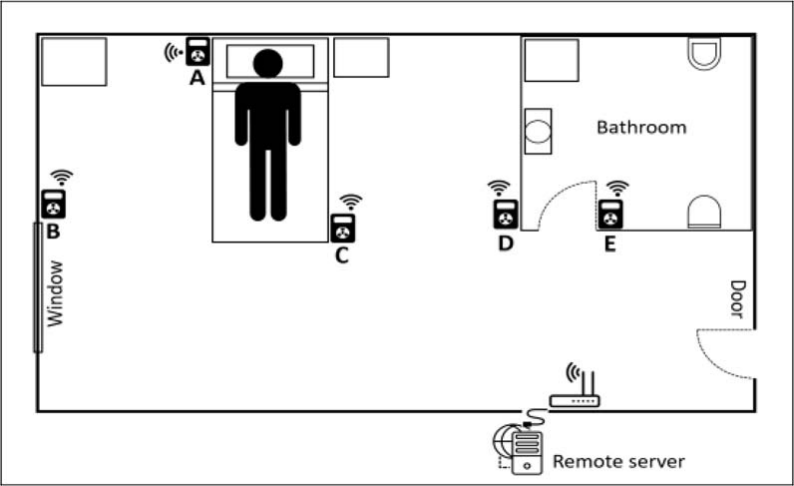
Position of the dosemeters relative to the patient in the shielded room: (A) 0.5 m; (B) 2 m; (C) 1 m; (D) 2 m; (E) in the bathroom.

In the first place, data on the dose to the operator in routine conditions, at different distances from the patient and 1 hour after radiopharmaceutical administration, were collected from the literature and measurement archives at the IFO and PMI facilities and given in Table 1. The experimental data refer to different patients and are given as a range from minimum to maximum dose rate. Dose rate computed with RESRAD-BUILD code for a patient after administration of ^131^I is also reported. The results of the simulation are in good agreement with the experimental data of the IFO and the other data shown in Table 1^(17–22)^. Nuclear medicine incidents may lead to higher radiation exposures of staff compared to routine procedures. These unintended events may include: handling of unsealed radionuclides or unshielded vials and syringes; malfunctioning of administration devices, with potential spillage of radioactive material; contamination from patient’s body fluids, such as where the patient vomits after swallowing a capsule of radio-iodine, or during the treatment of incontinent patients where leakage from a split urine bag may occur^(23, 24)^. It is clear from this list that incidents in therapy nuclear medicine shielded rooms are likely to happen during the delivery of therapy or patient assistance. Although there are articles describing incidents that may occur in NMT and bring potential and unintentional exposure to staff, no work has been found (to our knowledge) that provides the dose to the operators in these circumstances. Computational estimates is foreseen to be performed within this project. The system of detectors will be based on Geiger–Müller counters. The detectors will be placed in the shielded room with the configuration shown in Figure 3, mainly around the patient’s bed and in the bathroom. The choice is due to the expected higher dose rate levels in positions close to the patient, from a few centimeters up to 2 m away from the bed. One of the detectors is intended to be installed in the bathroom because, in this area, the surfaces that come into contact with the patient’s biological waste are expected to be more highly contaminated^(25)^.

**Table 1:** Ambient dose equivalent rate in a shielded room with a patient for different therapies

Radionuclide	Dose rate [μSv/h/GBq] at 1 hour after administration *vs* distance from patient [m]				Effective half-life (hours), *T*_eff_ Source	
	Contact	0.5	1	2		
^131^I			11.4–34.1		14.7–75.2	Measurements performed at IFO
		187	49	12		RESRAD-BUILD simulations
	610		50	15	13.9	(17;18)
^177^Lu DOTATATE					7.4–119.7	Measurements performed at IFO
	30	16	5	2.8	56	(19;20)
			1.1–2.4		64.1	Measurements performed at IFO
^90^Y glass microspheres	29–35	0.6–2.2	0.2–0.6		64.1	Measurements performed at PMI
	29		0.59		64.2	(21;22)

After confirming which areas are more concerned with radiation activity, the next step is to set up an IoT dosimetry system to provide a continuous and real-time radiation monitoring of each area of the room where the sensors are located and thus allowing a more effective identification of radiation-related events and reconstruction of the dose. To achieve this purpose, all the detectors shall communicate the measurement data via Wi-Fi to a web platform, working on a remote server that will be installed on the hospital network. This application is intended to centralise the management of several locations and provide online visualisation and analysis tools for the collected data, as well as data storage and export. Access to the application will be possible from any PC according to the security rules established by the hospital IT department. Wi-Fi coverage is an issue that should not be ignored when dealing with shielded rooms, as the shielded walls may prevent the outside signal from entering the room. In this case, the easiest solution is to put a Wi-Fi access point inside the room, provided that there is at least an ethernet socket to connect with the hospital network.

## Discussion

In this work, we proposed an incident reporting system designed for the NMT Unit. This IoT system is composed of an app for smartphones and a wireless network of detectors. The app is expected to meet the need for a little laborious reporting procedure that lightens the already heavy workload of the staff in the Unit. The network of detectors allows for a real time collection of the dose rate distribution in the patient’s room.

The face-to-face interviews turned out to be an effective tool for the identification of the needs and expectations of the staff with regard to an incident reporting system, although 24 operators obviously cannot be considered a representative sample. It was useful, however, to get a real overview of the attitude toward potential events and to identify the situations felt as the most critical by the staff. The operators showed interest and participation during the interviews and welcomed the invitation to be involved in the design of the system.

The setting up of the system in a small department proved to be a successful choice because it made the communication between the system developers and the health operators straightful and easy. As Stavropoulou *et al*.^(26)^ demonstrated, incident reporting can indeed be more effective if it is managed at the micro-level, i.e. by clinical teams or small units rather than centralised in the department or in the whole organisation. The way the staff reacted and contributed to the interviews confirmed these results.

From other studies, it is known that the number of events is lower when the staff is well aware of the procedures and when these are in place and known by everyone^(27)^. For this reason, an app could be a valuable system to make these procedures available at the right time in any circumstances.

One concern regarded whether the reporter should provide a list of personnel observing the scene because privacy issues were raised. However, a great deal of information can be gained by deep discussion with the involved staff. Ford and Evans^(28)^ have pointed out that discussing the event with the staff that was present at the event, either in a cooperative fashion or in a private interview, leads to rich and informative reconstruction of the event and helps to stress the importance of fact-finding for improving safety.

A system for dose rate detection was added, with several Geiger-Müller devices in fixed positions. These were located mainly close to the patient’s bed and in the bathroom, under the hypothesis that in NMT, the patient is the source, and therefore most events with a potential of exposure for the workers are expected to occur close to the patient. The fixed position of the detectors allows to reduce the uncertainties in the potential difference in dose rates between routine and accidental situations.

It must be noted that the radiation exposure of nurses and medical staff in routine operations, with hospitalised nuclear medicine patients, is typically on the order of a few tens of μSv/h, which is reflected in an annual dose on the order of 5 mSv^(12, 13)^. Any accident or abnormal event that may occur in the patient’s room (i.e. urine leakage from the catheter, vomiting or dropping of the iodine tablet) may result in deviations from the dose usually received by the operators, which is expected to be low. The reason for using a shielded room for this pilot project was to reduce the number of variables, with the same patient staying for several days, predictable paths and fluxes and a well-defined team to be involved.

Once the model will be consolidated, it could be improved by integrating the data collected by the app and the system of detectors. As the type of incident depends also on the characteristics of the patients^(7)^, it will be useful to integrate data on the patient and therapy, especially if these are available as bar or QR codes^(29)^. Tracking the operators’ position with wireless/bluetooth systems can add information for the reconstruction of both what happened and the dose to the worker.

Finally, the app has a section where the operator can provide a freely editable description of the event. Although this facilitates the storytelling in the style and language of the operator and is expected to be associated to a greater detail, it makes the insight and analysis of the data more difficult. An additional improvement could be done by using the textual reporting for the definition of a taxonomy that may help to classify the events according to type and severity, to propose resolution procedures, following what recommended by the Bonn Call for Action (‘Harmonize taxonomy in relation to medical radiation incidents and accidents, as well as related communication tools such as severity scales, and consider harmonization with safety taxonomy in other medical areas’)^(3)^.

## Conclusions

A system for internal and voluntary reporting of abnormal events in an NMT Unit was developed. The mobile application has great potential as a user-friendly tool for healthcare professionals aiming at making the incident reporting procedure little laborious. The detectors network allows for real-time measurement of the dose rate in the patients’ shielded room by using wireless communication. The involvement of the staff through interviews and social science approach has guided the design of the system since its initial phase and is expected to be an added value in the final testing of the product.

## Data Availability

The original contributions presented in the study are included in the article, further inquiries can be directed to the corresponding author.
